# N-3 polyunsaturated fatty acids may affect the course of COVID-19

**DOI:** 10.3389/fimmu.2022.957518

**Published:** 2022-09-27

**Authors:** Barbora Lampova, Ivo Doskocil, Lenka Kourimska, Aneta Kopec

**Affiliations:** ^1^ Department of Microbiology, Nutrition and Dietetics, Faculty of Agrobiology, Food and Natural Resources, Czech University of Life Sciences Prague, Prague, Czechia; ^2^ Department of Human Nutrition and Dietetics, Faculty of Food Technology, The University of Agriculture in Krakow, Krakow, Poland

**Keywords:** fatty acid, fish oil, inflammation, nutrition, SARS-CoV-2, bioactive metabolites, human health

## Abstract

The highly infectious coronavirus disease (COVID-19) is caused by severe acute respiratory syndrome coronavirus 2 (SARS-CoV-2), which is a new coronavirus that has been spreading since late 2019 and has caused millions of deaths worldwide. COVID-19 continues to spread rapidly worldwide despite high vaccination coverage; therefore, it is crucial to focus on prevention. Most patients experience only mild symptoms of COVID-19. However, in some cases, serious complications can develop mainly due to an exaggerated immune response; that is, a so-called cytokine storm, which can lead to acute respiratory distress syndrome, organ failure, or, in the worst cases, death. N-3 polyunsaturated fatty acids and their metabolites can modulate inflammatory responses, thus reducing the over-release of cytokines. It has been hypothesized that supplementation of n-3 polyunsaturated fatty acids could improve clinical outcomes in critically ill COVID-19 patients. Some clinical trials have shown that administering n-3 polyunsaturated fatty acids to critically ill patients can improve their health and shorten the duration of their stay in intensive care. However, previous clinical studies have some limitations; therefore, further studies are required to confirm these findings.

## Introduction

Coronavirus disease (COVID-19) caused by severe acute respiratory syndrome coronavirus 2 (SARS-CoV-2) is a highly infectious disease. The first known case was identified in Wuhan, China, in December 2019. The disease first spread in China and then worldwide, resulting in the World Health Organization (WHO) declaring COVID-19 a global pandemic on March 11, 2020 (1). Coronaviruses can infect animals and humans and affect the respiratory, central nervous, cardiovascular, or gastrointestinal systems (2, 3). The symptoms of COVID-19 include fatigue, muscle pain, headache, fever, dry cough, breathing difficulty, vomiting, diarrhea, and loss of taste and smell. However, many people do not experience any symptoms at all (4–6). Although approximately 80% of symptomatic individuals have a relatively mild disease course, 20% may develop acute respiratory distress syndrome (ARDS), sepsis, heart failure, or multi-organ dysfunction (7). The risk of severe disease increases with age and is higher in individuals with comorbidities such as hypertension, obesity, diabetes, metabolic syndrome, and chronic lung disease (8–11).

The first 2 weeks after the onset of COVID-19 are crucial (9, 11). Whether a patient develops severe complications depends on the initial viral load and innate immunity. The virus can be blocked in the upper airways and not reach the lungs. In this case, the patient has a much better chance of a mild course of infection. However, if the virus reaches the alveoli, it can replicate without local resistance (12). Due to a severe delayed adaptive immune response, there may be an excessive and uncontrolled release of pro-inflammatory cytokines, a so-called cytokine storm, leading to ARDS, cardiovascular damage, or multi-organ failure (7). The pro-inflammatory cytokine, interleukin 6 (IL-6) plays a key role in cytokine storms. Several studies have shown that serum levels of pro-inflammatory cytokines, particularly IL-6, are elevated in patients diagnosed with COVID-19. There is a strong correlation between mortality in patients with COVID-19 and the serum levels of pro-inflammatory cytokines. This suggests that an uncontrolled inflammatory response is a major factor in the adverse reactions observed in patients with COVID-19. Given these findings, minimizing the inflammatory response and reducing cytokine release could be a suitable preventive approach (13–15).

Immune responses to various infections, including virus infections, are strongly associated with nutritional state and lifestyle (16). Based on current knowledge, many nutrients, including proteins, essential fatty acids, vitamins, and minerals, play an important role in immune responses (16, 17).

Several studies have shown that n-3 polyunsaturated fatty acids (n-3 PUFAs) and their metabolites exhibit a range of biological effects that can modulate inflammatory responses and reduce cytokine release (18–20). These compounds may also affect viral entry and replication. Several clinical trials have provided evidence that adequate supplementation of n-3 PUFAs can improve clinical outcomes in patients with ARDS and sepsis, which are among the most common causes of death in critically ill patients with COVID-19 (21). Given these positive effects and their low-risk profile, it is appropriate to consider administering n-3 PUFAs as a supportive treatment for patients with COVID-19. From the outset of the pandemic, many authors published articles in which numerous nutritional compounds were described as important factors for the immune response. This review is focused on the effect of n-3 PUFAs in COVID-19 patients because of their well-documented beneficial effects on human health. It is difficult to find conclusive information in existing literature regarding the ideal dose of n-3 PUFAs in a regular diet or as a supplement taken during the SARS-CoV-2.

## Highly pathogenic human coronaviruses

Coronaviruses are coated RNA viruses belonging to the *Coronaviridae* family, which are named after their typical appearance. Prior to the severe acute respiratory syndrome (SARS) outbreak in 2002, these viruses were thought to cause mild upper respiratory tract infections in humans and rarely cause lower respiratory tract infections (22, 23).

A new human coronavirus, severe acute respiratory syndrome coronavirus 1 (SARS-CoV-1), the virus that causes SARS, was first identified in 2002 in China (24). It was later determined that SARS-CoV-1 was transmitted to humans, probably due to a mutation occurring in bats. However, this transmission is relatively inefficient because it requires direct contact with an infected individual (22).

In 2012, another new human coronavirus, Middle East respiratory syndrome coronavirus (MERS-CoV), was first identified in the Middle East, causing respiratory tract infections with a mortality rate of approximately 50% (25). However, it did not spread widely during the following year and it was brought under control by 2014. This virus is thought to have arisen from a mutation of a coronavirus occurring in a non-human host, with camels being the most likely natural hosts (22).

In December 2019, several cases of pneumonia of unknown origin were reported in Wuhan, China. The virus that causes this pneumonia showed phylogenetic similarities to MERS-CoV and SARS-CoV-1. The disease rapidly spread beyond China, which led the WHO to declare COVID-19 a public health emergency of international concern on January 30, 2020, and a pandemic on March 11, 2020 (1). Brief comparison of SARS-CoV-1, MERS-CoV, and SARS-CoV-2 is summarized in **Table 1**.

**Table 1:** Brief comparison of SARS-CoV-1, MERS-CoV, and SARS-CoV-2.

	SARS-CoV-1	MERS-CoV	SARS-CoV-2	References
Family	*Coronaviridae*	*Coronaviridae*	*Coronaviridae*	(26)
First outbreak	Shunde, China	Saudi Arabia	Wuhan City, China	(26)
First identification	2002	2012	2019	(26)
Major reservoir	Bats	Camels	Bats	(22)
Transmission	respiratory droplets, close contact with infected persons, aerosol	respiratory droplets, close contact with infected person or camel, consumption of raw milk or meat from infected camel	respiratory droplets, close contact with infected persons, touching or eating infected object	(27)
Case Fatality Rate	~ 10%	~ 36%	1.25% (till 5. April 2022)	(26)
Incubation Period	2-7 days	2-14 days	2-14 days	(28)
Common symptoms	fever, headache, nausea, vomiting, fatigue, dry cough, sore throat, diarrhea, myalgia	fever, cough, diarrhea, sore throat,	fever, cough, headache, nausea, vomiting, fatigue, myalgia, diarrhea, sore throat	(26)
Prevention	social distance, hand hygiene	hand hygiene, avoidance of consumption of raw camel milk or meat	social distance, hand hygiene, vaccination	(26, 29)

SARS-CoV-1, Severe acute respiratory syndrome coronavirus 1; MERS-CoV, Middle East respiratory syndrome coronavirus; SARS-CoV-2, Severe acute respiratory syndrome coronavirus 2.

## Pathogenesis of coronavirus disease

The spike (S) protein present in the viral envelope is key to the entry of SARS-CoV-2 into the host cell, ensuring that the virus attaches to the host cell (30, 31). Viral entry is mediated by the binding of S protein to the angiotensin-converting enzyme 2 (ACE2) receptor, which is strongly expressed on the surface of pulmonary and intestinal epithelial cells, explaining their susceptibility to SARS-CoV-2. ACE2 receptors are also present in a number of other tissues, such as the kidneys and heart (32). SARS-CoV-2 is much more easily transmitted than MERS or SARS-CoV-1, due to its approximately 10–20-fold greater affinity for ACE2 receptors. Increased affinity of the virus for the ACE2 receptor is also correlated with disease severity (33, 34). After binding of the S protein to the ACE2 receptor, there may be a phase of penetration where the virus enters the cell *via* endocytosis, where an increase in the flow of H^+^ into the endosome activates L-cathepsin, which activates the S protein and facilitates fusion of the viral membrane and the release of ssRNA from the endosome (35). Once viral RNA is found in the cytoplasm or endosomes, it is detected by pattern-recognition receptors, such as toll-like receptors, which in turn initiate a defense by the innate immune system. The intensity of the innate immune and inflammatory response varies depending on the type of virus and viral load and is also influenced by the immune state and the age of the host (36). Innate immune system cells first trigger the expression of interferon 1, followed by activation of nuclear factor kappa B and production of pro-inflammatory cytokines to eliminate viruses (21, 37, 38).

In general, cytokines play a key role in the immune response against viruses and bacteria. However, excessive and uncontrolled release of pro-inflammatory cytokines, known as a cytokine storm, can lead to necrosis or apoptosis of the affected cells (7, 39, 40). Cell damage can also lead to the release of damage-associated molecular patterns, which are molecules derived from the nuclei or cytosol of damaged cells. These damage-associated molecular patterns can be recognized and, when pattern-recognition receptors are activated, can stimulate the production of pro-inflammatory cytokines and chemokines, contributing to the intensification of the inflammatory response (41).

The inflammatory cascade is further fueled by increased blood vessel permeability, resulting in the accumulation of inflammatory monocytes, macrophages, and neutrophils in various organs. Monocytes, macrophages, and neutrophils have been shown to be major sources of pro-inflammatory chemokines and cytokines during COVID-19, and as the cytokine storm grows stronger, the regulation of the immune response is compromised, leading to severe consequences (40, 42). Infected immune cells can migrate in the body and can thus cause systemic dissemination of the virus, which also contributes to the overall clinical deterioration (43).

Although approximately 80% of symptomatic individuals have a relatively mild course of the disease, which may include fatigue, muscle pain, fever, loss of appetite, smell, or cough, approximately 20% of patients develop ARDS, sepsis, respiratory failure, heart failure, liver damage, kidney damage, or multi-organ dysfunction, which may lead to death (7, 44–47). Several scientific publications have shown that sepsis, ARDS, coagulopathy, heart and respiratory failure, or multi-organ failure are the most common causes of death in critically ill patients with COVID-19 (11, 48–50).

Patients with severe COVID-19 have been shown to have increased levels of cytokines such as interleukins IL-6, IL-7, IL-8, IL-9, IL-10, IL-1β, IL-1RA, fibroblast growth factor, platelet-derived growth factor, tumor necrosis factor-alpha (TNF-α), granulocyte-macrophage colony-stimulating factor, monocyte chemoattractant protein, and vascular endothelial growth factor (8, 9, 51, 52). A significant finding is that there is a strong correlation between mortality in COVID-19 patients and elevated serum pro-inflammatory cytokine levels (3).

In addition, one study found that the median time from onset of symptoms to hospital admission, ARDS, and intensive care unit (ICU) admission was 7 days, 9 days, and 10.5 days, respectively (9). A worsening clinical condition with decreasing viral load and a gradual increase in pro-inflammatory cytokine levels were also observed in patients with severe COVID-19, suggesting that severe lung damage in COVID-19 patients is largely due to the immune response, rather than due to the direct effects of infection. This suggests that an uncontrolled inflammatory response is a major factor in the adverse reactions observed in COVID-19 patients. Given these findings (3, 7), minimizing the inflammatory response and reducing cytokine release could be an effective prevention strategy.

**Figure 1:**
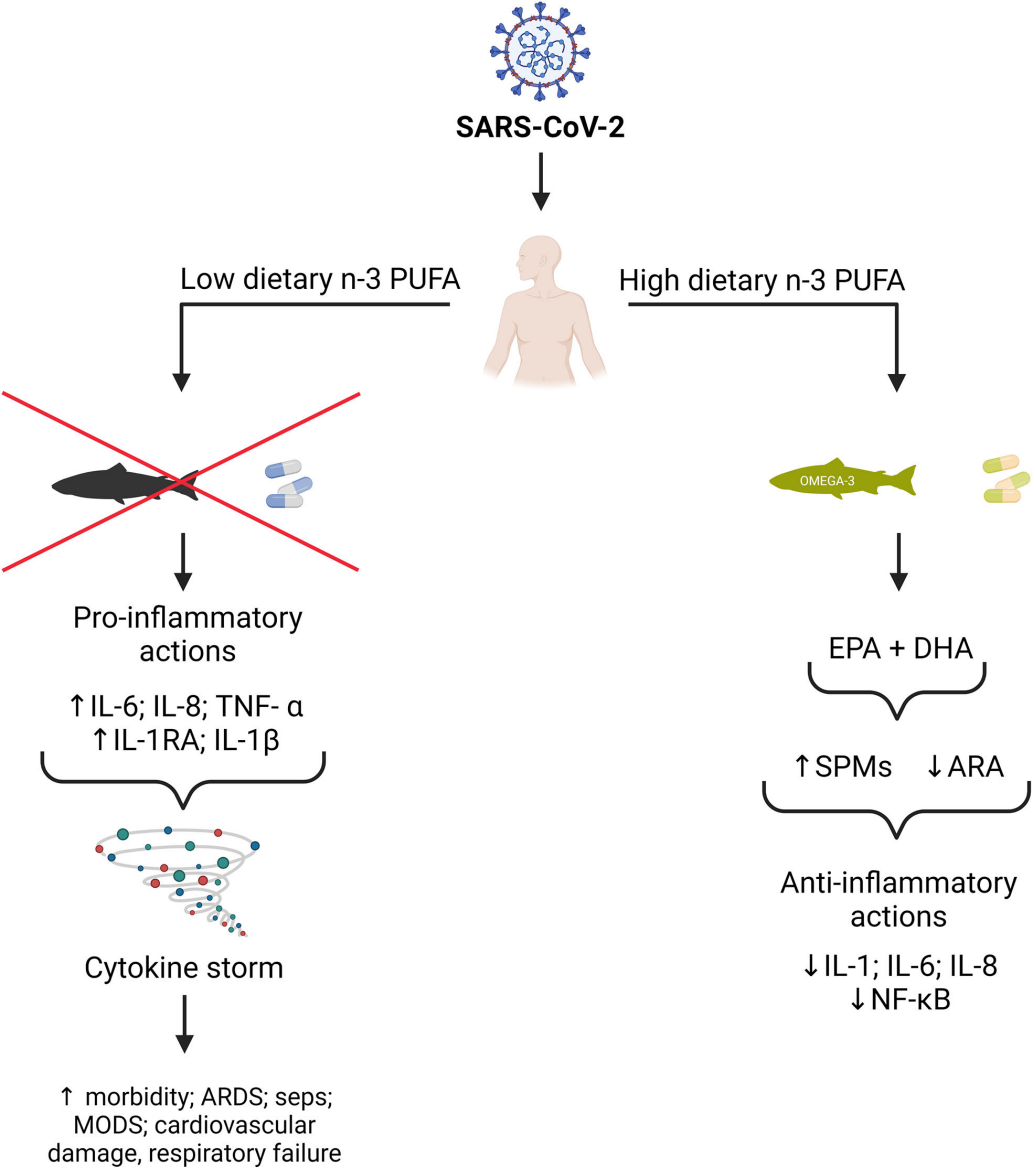
Potential benefits of n-3 PUFA on SARS-CoV-2 outcomes. EPA and DHA from dietary sources and supplements can replace the pro-inflammatory arachidonic acid (ARA) in cell membranes. EPA and DHA can also be metabolized to specialized pro-resolving mediators (SPMs), which inhibit the synthesis of pro-inflammatory cytokines such as IL-1, IL-6, and IL-8 *via* down-regulation of the kappa B nuclear factor pathway. (This figure was created by BioRender.com software).

Because n-3 PUFAs and their metabolites exhibit a variety of biological effects that can modulate inflammatory responses and the release of cytokines (**Figure 1**), and also interact at different stages of viral infection, especially in viral entry and replication, the benefits of n-3 PUFA supplementation in people with COVID-19 is the subject of current research. Currently, several clinical trials have shown that supplementation of n-3 PUFAs in adequate doses can improve the clinical outcomes in critically ill patients (7, 18–21).

### N-3 polyunsaturated fatty acids

N-3 PUFAs include α-linolenic acid (ALA, C18:3 n-3), eicosapentaenoic acid (EPA, C20:5 n-3), and docosahexaenoic acid (DHA, C22:6 n-3) (53). These fatty acids are considered essential because the simplest n-3 PUFA, ALA, cannot be synthesized *de novo* in mammals, due to the absence of the necessary enzymes. ALA can be partially transformed in the body into EPA and DHA by elongation and desaturation. As these compounds are considered essential, they must be obtained through the diet (54). For humans, the most important source of EPA and DHA is fish, such as anchovy, herring, mackerel, mullet, sardines, salmon, sturgeon, tuna, and trout (55). EPA and DHA are not only found in fish oil but are also present in seaweed; however, these compounds are present in much smaller amounts in algae (56). Unlike EPA and DHA, foods of plant origin with a high fat content are a source of ALA, particularly flax seeds, walnuts, rapeseeds, and quinoa (57).

Based on current knowledge, the range of the stipulated intake of n-3 fatty acids is 0.5–2% of total energy, and the minimum requirement of EPA + DHA is 250 mg/day for adults (58). This adequate intake of omega-3 fatty acids is ensured by consuming at least two portions of fish per week. In the case of pregnant and lactating women, the stipulated intake of DHA + EPA is 0.3 g/day and at least 0.2 g/day of DHA is stipulated to be consumed (58–62). In the case of children, adequate intake is dependent on age. For example, for infants aged 0–6 months, ALA accounts for 0.1–0.18% energy. The ALA requirement for children aged 6–12 months is 10–12 mg/kg (61).

However, a vast majority of the population in many countries, including the Czech Republic and Poland, does not regularly consume fatty fish and therefore, does not receive sufficient amounts of omega-3 fatty acids (63). Insufficient consumption of fish is caused by many factors, such as aversion to fish, costs, alternative diets that do not allow the consumption of fish, or concerns about contaminants such as mercury. In case of insufficient fish consumption, intake of omega-3 fatty acids through dietary supplements is recommended (57). Harton et al. (2013) reported that the intake of n-3 in the daily diet of pregnant women was too low. The average intake of EPA + DHA was 122 ± 205 mg/day and that of DHA was 87.1 ± 126 mg/day. Nowacka et al. (64) have also reported that the daily diet of canoeists and sport shooters was deficient in n-3 fatty acids. Sioen et al. (62), in an overview of n-3 intake in European countries, stated that there is a paucity of literature on this topic.

N-3 PUFAs have been studied for several years because of their widespread positive effects on human health. They improve cardiovascular health, brain function, and mental health. They also have a clinically significant positive effect on inflammatory diseases (56, 65).

N-3 PUFAs belong to a group of food supplements known as „generally regarded as safe”, meaning they are considered safe and only rarely cause mild side effects when consumed at the recommended dose. The side effects include an unpleasant fishy taste, bad breath, a change in body odor, and mild gastrointestinal discomfort accompanied by nausea or increased stool frequency. However, these side effects are generally mild and resolve spontaneously after discontinuation of n-3 PUFAs (27, 66).

As n-3 PUFAs reduce inflammatory reactions, which are very common in critically ill patients diagnosed with COVID-19 and may also affect virus replication, it is predictable that administration of an appropriate amount of n-3 PUFAs may accelerate recovery in COVID-19 patients (3, 21). N-3 PUFAs can act preventively against viral diseases, unlike anti-inflammatory drugs, which are already used during treatment and cannot be used as prophylactics. In addition, we can regulate the number of fatty acids used before and during the disease itself, by adjusting the diet to one rich in n-3 PUFAs. Currently, n-3 PUFA intake in Western countries is considerably lower than the recommended intake (21).

### N-3 polyunsaturated fatty acids can affect viral replication by influencing sterol regulatory element-binding protein

Fatty acids, including n-3 PUFAs, have a strong effect on many aspects of virus biology. In general, they can modulate changes in membrane composition which interferes with the accessibility of different viral receptors, changes the activity of membrane-linked enzymes, and modifies membrane ion transport. N-3 PUFAs specifically modulate the formation of membrane rafts (67) in which SARS-CoV-2 receptor (ACE2) and coreceptor (TMPRSS2) are located and therefore can decrease the level of interaction between the virus and the host cell (68). N-3 PUFAs can also modulate antiviral and proinflammatory responses *via* modulation of CD8+ T cell activity (69).

Viruses use host metabolic mechanisms to facilitate their replication. To obtain sufficient lipids for their replication, they manipulate the lipid metabolism of the host (21). This metabolic reprogramming is mediated by sterol regulatory element-binding protein (SREBP) transcription factor isomers. For example, to ensure MERS-CoV reproduction, the virus modifies the host cellular lipid metabolism and reprograms the *de novo* SREBP-dependent lipogenesis pathway (21, 70). MERS-CoV non-structural proteins nsp3 and nsp4 are used by the virus to reprogram cellular pathways leading to the formation of double-membrane vesicles (DMVs). DMVs are crucial cellular organelles necessary for replication of MERS-CoV as they provide the anchoring scaffold for viral replication and transcription complexes leading to the formation of so-called viral factories. The formation of DMVs may be disrupted by the blockade of fatty acid synthesis pathways. For example, AM580, an inhibitor of SREBP, also effectively inhibits replication of MERS-CoV *via* blocking of DMV formation (21, 70).

Three SREBPs have been identified: SREBP1a and SREBP1c, which regulate fatty acid metabolism genes, and SREBP2, which regulates cholesterol metabolism genes. SREBPs are regulated post-transcriptionally, and their inactive precursor form is localized in the endoplasmic reticulum membrane, where it is associated with SREBP cleavage-activating protein (SCAP) (71, 72). When sterols are abundant, SREBP is maintained by binding to regulatory SCAP. The SREBP/SCAP complex is anchored by insulin-regulated protein (Insig). As sterols in the intracellular space drop, Insig dissociates from the SREBP/SCAP complex. The SREBP/SCAP complex is subsequently translocated to the Golgi complex, where it undergoes proteolytic cleavage to produce a transcriptionally active amino-terminal fragment of SREBP called n-SREBP (73, 74).

Several research laboratories have demonstrated that n-3 PUFAs significantly reduce the concentration of transcriptionally active n-SREBP. The ability of n-3 PUFAs to decrease the concentration of transcriptionally active n-SREBP is probably due to both their physical and biochemical effects (73, 75–77).

Using large and small unilamellar vesicles as a model for plasma and intracellular membranes, Johnson et al. (78) found that when n-3 PUFA was added to these model membranes, cholesterol affinity for phospholipids decreased, resulting in increased cholesterol transfer from cholesterol-rich regions (such as the plasma membrane) to cholesterol-poor areas (such as endoplasmic reticulum). Another mechanism by which n-3 PUFAs decrease the concentration of transcriptionally active n-SREBP is by changing the composition of cell membranes. N-3 PUFAs can increase sphingomyelin hydrolysis. Sphingomyelin, which is present in plasma membranes, is hydrolyzed to ceramide and phosphocholine. The presence of lower amounts of sphingomyelin results in reduced ability to absorb free cholesterol, resulting in a decrease in SREBP-mediated gene transcription (73, 79–81). Ceramide has also been shown to be a potential inhibitor of SREBP (79).

### Effect of n-3 polyunsaturated fatty acids on inflammation

Possibly the best-known feature of n-3 PUFAs is their ability to reduce inflammation and their beneficial effect on inflammatory-related disorders. N-3 PUFAs can be incorporated into phospholipid membranes, where they can replace n-6 PUFAs (56). Most cells contain relatively large amounts of arachidonic acid (ARA, C20:4 n:6) in their cell membranes compared with other polyunsaturated fatty acids (82). These 20-carbon fatty acids with four double bonds can be released from the cell membrane by phospholipase A2 (83). ARA can be metabolized to several pro-inflammatory eicosanoids by the action of lipoxygenase (LOX), cyclooxygenase (COX), and epoxygenase P-450 enzymes. For example, COX catalyzes the conversion of ARA to prostaglandin G2 and subsequently to prostaglandin H2, which is a substrate for the production of other prostaglandins (prostaglandin D2, prostaglandin E2, and prostaglandin F2α), thromboxanes (thromboxane A2 and thromboxane B2), and prostacyclin. These compounds are collectively called prostanoids. LOX first metabolizes ARA to the corresponding hydroperoxyeicosatetraenoic acids, which are further metabolized to the corresponding hydroxyeicosatetraenoic acids, from which leukotrienes (leukotriene B4, leukotriene C4, leukotriene D4, and leukotriene E43) are synthesized (3, 84). N-3 PUFAs may compete with ARA for the same metabolic enzymes and thus become preferential substrates, resulting in the production of fewer pro-inflammatory and anti-inflammatory metabolites. Increasing the n-3 PUFA content in cell membranes can therefore reduce inflammatory responses (3).

The benefits of n-3 PUFA supplementation were confirmed in a study conducted by Gerling et al. (85), which examined the effect of n-3 PUFA supplementation on the n-6/n-3 PUFA ratio in the muscle and mitochondrial membranes of healthy men. Consuming 3 g of EPA + 2 g of DHA per day for 12 weeks increased the EPA and DHA content of the membranes and consequently decreased the n-6/n-3 PUFA ratio. These results suggest that n-3 PUFA supplementation may reduce the availability of ARA for the synthesis of pro-inflammatory eicosanoids, and thereby reduce inflammatory reactions.

N-3 PUFAs can also be metabolized into high specialized pro-resolving mediators (SPMs), including protectins, maresins, resolvins, and lipoxins. These biologically active compounds are formed by the action of COX, LOX, and cytochrome P450 on EPA and DHA. SPMs show strong anti-inflammatory effects that are necessary to end the inflammatory processes in the body, accelerating tissue regeneration and tissue repair (86, 87). SPMs can inhibit the synthesis of pro-inflammatory cytokines such as IL-1, IL-6, and IL-8 *via* down-regulation of the kappa B nuclear factor pathway (88). EPA-derived resolvins (E-series resolvins), DHA-derived resolvins (D-series resolvins), and DHA-derived protectins exhibit anti-inflammatory effects by limiting leukocyte infiltration into damaged tissues (89, 90). Maresins, which are synthesized from DHA, stimulate macrophagocytosis and reduce neutrophil infiltration into damaged tissue (91). 13(S),14(S)-epoxymaresin also inhibits the production of a pro-inflammatory cell mediator derived from ARA through direct inactivation of the leukotriene-A4 hydrolase enzyme, which catalyzes the conversion of leukotriene A4 into the pro-inflammatory metabolite, LTB4 (86). SPMs also promote the removal of apoptotic cellular debris from inflamed tissue and limit the formation of free radicals (92).

The effect of n-3 PUFA on inflammatory status has been evaluated in obese patients diagnosed with diabetes. After 8 weeks of consuming 1.44 g/day of EPA + 0.96 g/day of DHA, an overall improvement in insulin sensitivity and reduction in inflammatory markers such as IL-1β, IL-6, and TNF-α was observed (93). Improvements in the inflammatory status were also observed in a study conducted by Moghadam et al. (94), which assessed the effect of consuming 1.548 g/day of EPA + 0.828 g/day of DHA + 0.338 g/day of n-3 PUFA in patients diagnosed with type 2 diabetes. After 8 weeks of consumption, an overall decrease in the blood concentrations of IL-2 and TNF-α was observed.

The anti-inflammatory effects of high doses of n-3 PUFA (1.5 g/day EPA + 1.0 g/day DHA for 4 weeks) were also confirmed in a randomized controlled trial in which significant reductions in plasma levels of IL-6, IL-1β, and TNF-α were observed in patients with chronic venous ulcers in their lower legs. The results of this study also suggest that n-3 PUFAs may affect the major components of a cytokine storm (95).

Several studies have examined the effects of SPMs on viral diseases. Morita et al. (96) investigated the effects of protectin D1 on the course of influenza caused by the H5N1 virus in mice. This study found that protectin D1 suppressed influenza virus replication, and its administration improved survival and disease pathology in mice. Ramon et al. (97) investigated the effects of 17-hydroxydocosahexaenoic acid on the adaptive immune response in mice infected with the H1N1 influenza virus. DHA-derived SPM 17-hydroxydocosahexaenoic acid increased antibody levels, leading to greater resistance to the H1N1 influenza virus. Moreover, DHA pretreatment of SH-SY5Y cells infected with Zika virus has been reported to increase cell viability, reduce apoptotic cells, increase the proliferative capacity of infected cells, significantly reduce viral load, and reduce the secretion of proinflammatory cytokines IL6 and monocyte chemoattractant protein-1, thereby alleviating the pro-inflammatory response caused by Zika virus (98).

### Effect of n-3 polyunsaturated fatty acids on acute respiratory distress syndrome

Patients with severe COVID-19 often develop ARDS. ARDS is caused by a dysregulated inflammatory response in the lung tissue or a cytokine storm (99, 100). During the inflammatory response, the pulmonary capillaries and alveoli are damaged, causing increased permeability to fluid and proteins, leading to fluid accumulation in the lungs. Consequently, there is an increase in the number of inflammatory cells in the lung tissue and alveoli, a reduction in pulmonary pliability, pulmonary hypertension, and prolongation of the oxygen diffusion pathways. ARDS manifests as dyspnea, rapid breathing, and hypoxemia (101, 102). Patients with severe ARDS are at increased risk of developing sepsis and cardiac arrest (9). ARDS can lead to the failure of other vital organs, which may lead to death (103). According to a meta-analysis conducted by Singer et al. (104), which included 10815 patients diagnosed with ARDS, the overall mortality was 39%.

Several studies have evaluated the effects of consuming EPA, gamma-linolenic acid (GLA), and antioxidants on ARDS outcomes. Singer et al. (105) found that in 100 patients diagnosed with ARDS, consuming an enteral diet enriched with EPA, GLA, and antioxidants for 14 days resulted in significant improvements in oxygenation and reduced the duration of mechanical ventilation. A meta-analysis conducted by Pontes-Arruda et al. (106), found that administration of enteral nutrition enriched with EPA, GLA, and antioxidants to mechanically ventilated patients with acute lung injuries or ARDS significantly reduced their mortality, incidence of new organ failure, duration of mechanical ventilation, and duration of ICU stay. Moreover, a study conducted by Shirai et al. (107) found that the administration of an EPA, GLA, and antioxidant-containing enteral diet to critically ill patients with ARDS shortened the duration of their ICU stay. However, this study did not find a reduction in the need for mechanical ventilation or reduced mortality. A randomized double-blind study (108) performed on 58 adult patients with mild to moderate ARDS showed that the administration of n-3 PUFA in the form of 720 mg n-3 PUFA capsules containing 360 mg EPA and 240 mg DHA thrice daily led to improvements in certain parameters, such as indices of lung mechanics and oxygen partial pressure. Both groups received pantoprazole for prophylaxis against stress ulcers prophylaxis and heparin for prophylaxis against deep vein thrombosis. However, there was no statistically significant difference in the length of ICU stay between the intervention and the control groups.

A meta-analysis by Langlois et al. (19) found that the consumption of n-3 PUFAs, GLAs, and antioxidants can improve the exchange of lung gases, leading to a shorter ICU stay in critically ill and a shortened duration of mechanical ventilation in critically ill patients with ARDS, although significant heterogeneity was found in the individual studies and most of the studies showing positive effects were published prior to 2011. Langlois et al. (19) stressed that the effects of n-3 PUFAs, GLA, and antioxidants are dependent on the route and method of administration.

In summary, it can be said that the consumption of n-3 PUFAs could provide certain benefits during the treatment of patients with ARDS, such as shortening stays in ICUs, improving oxygenation, reducing the risk of death, and reducing the duration of mechanical ventilation. However, to definitively confirm these positive effects, further and more extensive studies on COVID-19 patients are needed.

### Effect of n-3 polyunsaturated fatty acids on sepsis

Sepsis is caused by the host’s dysregulated immune response to infection, which can lead to the onset of systemic inflammatory response syndrome (SIRS). SIRS refers to a condition in which at least two of the following criteria are met: hypothermia or fever (body temperature < 36°C or > 38°C), tachypnea (respiratory rate > 20 breaths/min) or CO_2_ < 32 mmHg, tachycardia (heart rate > 90 bpm), and abnormal leukocyte counts (leukocytes >12,000/µl or < 4,000/µl or > 10% immature forms) (109).

A study by Pontes-Arruda et al. (110) investigated the effect of administering an enteral diet enriched with EPA, GLA, and vitamins with antioxidant effects for 28 days in patients with severe sepsis or septic shock who required mechanical ventilation. The study found that diets enriched with EPA, GLA, and antioxidant vitamins were associated with more favorable outcomes, including lower mortality and a reduced duration of ICU stay.

Hosny et al. (111) conducted a study of the effects of short-term high-dose n-3 PUFA supplementation. The study results showed that short-term administration of high-dose n-3 PUFAs (9 g/day) to patients with early sepsis without associated organ dysfunction appeared to be safe and may have a beneficial effect. Intervention group had significantly lower levels of C-reactive protein (CRP), IL6, and procalcitonin than those in the control group, were less likely to require mechanical ventilation, had a shorter duration of mechanical ventilation (in cases where it was required), and were significantly less likely to develop severe sepsis, but did not experience a significant reduction in the 28-day mortality rate.

In their correlation analysis, which included 25 randomized controlled trials, Chen et al. (112) examined the effect of n-3 PUFA administration as part of enteral or parenteral nutrition on mortality in adult patients of various ages diagnosed with sepsis. In this review, it was found that supplementation with n-3 PUFAs as part of enteral or parenteral nutrition could reduce sepsis and ARDS-induced mortality. A meta-analysis conducted by Wang et al. (113) which included 20 suitable randomized clinical trials comprising a total of 1514 patients with sepsis indicated a possible reduction in mortality in patients with sepsis after administration of n-3 PUFA, shortening of the length of ICU stay, and a reduced need for mechanical ventilation. In a subgroup analysis, n-3 PUFAs were found to be positive, especially in patients with sepsis and gastrointestinal dysfunction. However, the authors of the study also pointed out that the improvement of certain parameters could also be caused by the addition of other nutrients, such as antioxidant vitamins and amino acids (arginine or glutamine), which can be added to enteral mixtures. Moreover, a meta-analysis conducted by Mo et al. (114), which included 12 studies and a total of 721 patients with sepsis who received n-3 PUFAs as part of parenteral nutrition, concluded that parenteral nutrition enriched with n-3 PUFAs may be beneficial for patients with sepsis. However, the authors have stated that these data should be interpreted with caution.

In conclusion, it can be said that n-3 PUFAs could show beneficial effects in sepsis, such as a reduction in mortality, shorter stays in ICUs, and shortened durations of mechanical ventilation. However, to definitively confirm these positive effects, more extensive studies on COVID-19 patients are required.

### Clinical studies on the effect of n-3 polyunsaturated fatty acids supplementation in COVID-19 patients

As the positive effects of n-3 PUFA supplementation on the key components of the cytokine storm, which is likely to be a major factor leading to a more severe clinical course of COVID-19, have been demonstrated previously, several randomized controlled trials have been conducted to examine the effect of n-3 PUFA supplementation on overall outcomes in patients with COVID-19.

A randomized, double-blind clinical trial examined the effect of n-3 PUFA supplementation on clinical and biochemical parameters in critically ill COVID-19 patients whose symptoms included severe pneumonia, fever, fatigue, dry cough, and ARDS. The study included 101 patients who were divided into intervention (28 patients) and control (73 patients) groups. Both groups received hydroxychloroquine. The intervention group was administered 400 mg EPA and 200 mg DHA daily for 14 days as part of enteral nutrition. EPA and DHA supplementation was associated with improved 1-month survival and had a positive effect on respiratory and metabolic acidosis (improved arterial pH, bicarbonate concentration, and base excess) and renal function (115).

In another randomized clinical trial involving 30 adults hospitalized with COVID-19, the effect of 2 g EPA + DHA supplementation on inflammatory reactions and clinical symptoms of the disease was investigated. N-3 PUFA supplementation has been found to improve some clinical signs of COVID-19, such as reduced body pain, fatigue, and increased appetite. Significant improvements in inflammatory markers, such as the serum erythrocyte sedimentation rate and serum C-reactive protein, were observed; however, n-3 PUFA supplementation did not improve olfactory dysfunction or affect circulating liver enzyme levels (116).

A pilot study of 100 patients with confirmed COVID-19 examined the relationship between the tissue levels of n-3 PUFAs and the risk of death. Patients with a higher n-3 PUFA index had a lower risk of death, although this result was not statistically significant (7).

An open-label randomized controlled trial is currently being conducted, to investigate the effect of n-3 PUFA supplementation (EPA 2 g/day) in hospitalized COVID-19 patients. The aim of the study is to clarify the effect of n-3 PUFA supplementation on oxygen saturation, IL-6 levels, mortality, duration of ICU stay, duration and need for mechanical ventilation, and overall duration of hospitalization (117).

Although these studies suggest that PUFA supplementation has potential benefits in patients with COVID-19, additional larger studies are needed to confirm the benefits of PUFA supplementation in patients with COVID-19, given the limitations of previous studies, such as small sample sizes and short study duration.

### Appropriate dosage of n-3 polyunsaturated fatty acids for adjunctive therapy in patients with COVID-19

When considering the inclusion of n-3 PUFA as adjunctive therapy in patients with COVID-19, it is important to determine the appropriate time to initiate treatment, the duration of treatment, route of administration, and overall formulation. The literature suggests that it may take several weeks for n-3 PUFAs to show a biological effect due to the time taken to replace ARA in cell membranes when receiving a normal dose (3).

It has been hypothesized that acute n-3 PUFA supplementation may influence the inflammatory response in critically ill patients. This hypothesis has been confirmed by a number of studies in patients with inflammatory diseases such as ARDS or sepsis even before the outbreak of the COVID-19 pandemic. However, the literature is not entirely consistent with regard to the effective dose at which anti-inflammatory effects are manifested.

According to Calder (118), the anti-inflammatory effects of n-3 PUFA manifest only when at least 2 g of EPA + DHA are consumed daily. The intake of such an amount of n-3 PUFA can be achieved by eating approximately one portion of fatty fish per day. However, fish intake is well below current recommendations in most Western countries, so most people in Western countries consume less than this amount.

According to Bistrian (119), mild anti-inflammatory effects can be achieved by consuming approximately 1 g of n-3 PUFA per day. Stronger effects on pro-inflammatory cytokine secretion can only be achieved by consuming larger amounts of n-3 PUFA, in the range of 4–6 g/day. However, the intake of such an amount of n-3 PUFA cannot be obtained from dietary sources alone and requires supplemental intake in enteral or parenteral form (118, 120). In addition, when n-3 PUFA is administered parenterally, there is no loss during the digestion and absorption process (121).

Acute administration of higher doses of n-3 PUFA could potentially lead to a significant improvement in clinical outcomes in patients with severe COVID-19. However, based on the evidence from the currently available clinical trials, it is not possible to reliably determine the most appropriate amount of n-3 PUFA or route of administration for patients with COVID-19 (3). For that reason, it would be advisable to carry out a larger study aimed at finding out the levels at which doses of n-3 PUFA manifest anti-inflammatory effects in COVID-19 positive patients.

## Conclusion

Despite the availability of COVID-19 vaccines, COVID-19 is still spreading rapidly worldwide; therefore, it is very important to place a greater emphasis on prevention.

There is evidence that administering n-3 PUFA as an adjunctive therapy in patients with COVID-19 has potential benefits. Previous studies have confirmed the beneficial effects of n-3 PUFA administration on the inflammatory state and cytokine storms. However, most of these studies were conducted before 2019 and were therefore not conducted in patients with COVID-19. The available evidence suggests that consuming an adequate amount of n-3 PUFA could improve the recovery of critically ill patients with COVID-19, shorten their ICU stay, reduce the need for mechanical ventilation, and generally accelerate recovery. Recent studies in patients with COVID-19 have shown a similar trend. The limitation of these studies were usually a short duration of experiment and a small number of participants. However, to confirm the beneficial effects of n-3 PUFA on the course of COVID-19, further larger studies are required.

## Author contributions

ID supervised, conceptualized, and edited the article. BL conceptualized and wrote the article. LK and AK made substantial contributions to the writing of the article and the overall editing. All co-authors approved the submitted version.

## Funding

This research was funded by the Grant Agency of the Czech Republic, grant no. GA 21-42021L. The METROFOOD-CZ research infrastructure project (MEYS Grant No.: LM2018100) includes access to its facilities and the research was funded by the National Science Centre Poland, grant no. 2020/39/I/NZ9/02959.

## Acknowledgments

We would like to thank Dr. Jiri Cerny and Prof. Milan Marounek for reviewing the manuscript.

## Conflict of interest

The authors declare that the research was conducted in the absence of any commercial or financial relationships that could be construed as a potential conflict of interest.

## Publisher’s note

All claims expressed in this article are solely those of the authors and do not necessarily represent those of their affiliated organizations, or those of the publisher, the editors and the reviewers. Any product that may be evaluated in this article, or claim that may be made by its manufacturer, is not guaranteed or endorsed by the publisher.
